# Dengue: a growing threat requiring vaccine development for disease prevention

**DOI:** 10.1080/20477724.2018.1514136

**Published:** 2018-09-14

**Authors:** Sandra Bos, Gilles Gadea, Philippe Despres

**Affiliations:** Unité Mixte Processus Infectieux en Milieu Insulaire Tropical, Plateforme Technologique CYROI, Université de La Réunion, INSERM U1187, CNRS UMR 9192, IRD UMR 249, Sainte-Clotilde, La Réunion, France

**Keywords:** dengue, epidemiology, clinical disease, antiviral immunity, prophylaxis, vaccine strategies, live-attenuated viruses, biological markers of dengue disease

## Abstract

Dengue disease is the most prevalent mosquito-borne viral infection in humans. At least one half of the global population is estimated at risk of infection and an estimated 390 million people are infected each year. Over the past few years, dengue burden continued to increase, mainly impacting developing countries. Alarming changes in dengue epidemiology were observed highlighting a spread from tropical to subtropical regions as well as urban to rural areas. An increase in the co-infections with the four serotypes has also been noticed, involving a shift in the targeted population from pediatric to adult. Facing these global changes, authorities will have to reinforce preventive actions and adapt healthcare management. New prophylactic strategies are urgently needed to prevent severe forms of dengue disease. The lack of specific antiviral therapies available turns vaccine development into a socio-economic challenge. In this review, we propose an update on the dengue global trends and different vaccine strategies in development. A particular attention will be paid to up-to-date information on dengue transmission and the protective efficacy of newly commercialized tetravalent dengue vaccine Dengvaxia®, as well as the most advanced candidate vaccines in clinical development.

## Introduction

Dengue virus circulates in many parts of the world, impacting most tropical and subtropical countries. Millions of people are affected each year and global dengue incidence has dramatically increased in recent decades. Dengue fever is a flu-like illness that usually heals after three to seven days. However, dengue disease sometimes causes life-threatening complications. Although dengue disease has been twice classified by the World Health Organization (WHO) in 1997 and in 2009, severe disease prediction and monitoring still remain unsatisfactory. In addition, the burden of dengue disease represents a real threat to affected countries, some of which are facing economic difficulties. An efficient prophylactic vaccine strategy is urgently needed to tackle dengue infections worldwide. We hope that this work, by reviewing the global trends of dengue virus epidemiology, biology, and clinical disease, will help to better understand current vaccination strategies.

## Dengue disease

### Epidemiology of dengue

Since its discovery in 1779, dengue disease rapidly evolved into a major public health problem which still remains present today. Dengue is now the most medically-important arthropod-borne viral infection with nearly one-half of the world population at risk. Dengue virus (DENV) is predominantly transmitted by *Aedes (Ae.) aegypti*, and to a lesser extent, by *Ae. albopictus* mosquitoes [1]. Over the past 50 years, the global dengue incidence dramatically increased to almost 30 fold, reinforcing an already high economic burden causing both human suffering and massive socioeconomic losses [2]. Such figures underscore the failure of implemented procedures to control virus transmission and accentuate the need of adapted vaccine development.

**Figure 1.**
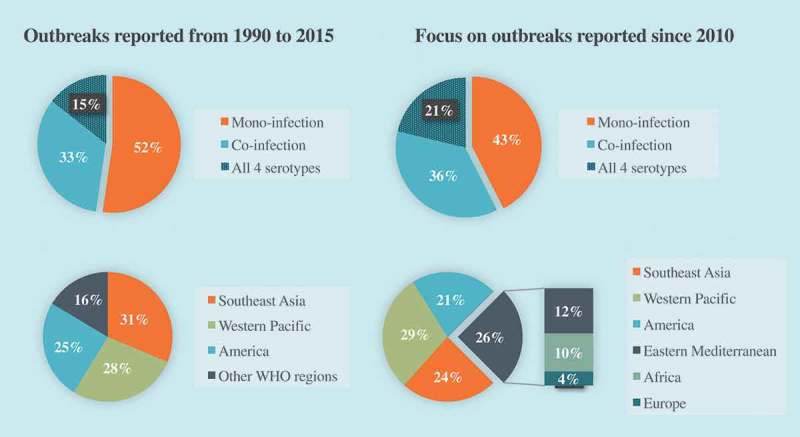
Evolution of epidemiological trends of outbreaks from 1990 to 2015. Dengue serotypes distribution: data based on 174 outbreaks including 80 from 2010 (46%) outbreaks distribution among WHO regions: data based on 262 outbreaks including 112 from 2010 (43%) (data from GUO et al, 2017).

According to the WHO, the actual numbers of dengue cases are underreported and 3.9 billion people, representing half of the global population, are estimated at risk of infection [3]. One recent estimate indicates 390 million dengue infections per year (95% credible interval 284–528 million) instead of the 50 to 100 million cases usually quoted, of which 96 million (67–136 million) manifest clinically, independently of the severity of disease [4]. The geographic range of DENV widely spread from tropical to most subtropical regions where endemicity was facilitated by the abundance of vectors and high population density. Most hyperendemic regions are located in Asia and Latin America, with a particular focus towards Thailand, Philippines, India and Brazil which experienced the highest number of severe outbreaks in the last decades [2]. In recent years, outbreak reports and meta-analysis raised up changes in epidemiological trends of the disease (Figure 1).

Although DENV is still persistent and mostly transmitted in urban areas, outbreaks in rural areas are becoming more and more frequent suggesting that dengue is not confined to urban areas anymore. The expansion to rural areas has been especially spotted in Southeast Asia and India where the prevalence of dengue was rising so fast that it has converged towards the high rates detected in urban populations [5]. As villages develop without proper water supplies, inhabitants use water storage vessels that provide breeding sites for the mosquitoes causing dengue fever. Moreover, urbanization prompts persons from rural areas into frequent travel between large cities and relatives in their villages. This way, growing interactions attenuate boundaries from both areas and enhance the risk of transmission. This finding underscores the urgent need to create awareness among the rural population and to conduct education programs providing good habits and behavior that help vector control.

As there are not yet specific medications to treat severe dengue, prevention remains the most important action to reduce the risk of dengue infection (WHO guidelines 2009). Two major ways to prevent dengue infection are efficiently experienced: mosquito control by either larval or adult control, and reducing mosquito bites especially during daylight hours. People experiencing fever from DENV infection should not be in an environment where they may be bitten by mosquitoes. If this is not possible, they should stay at home until they have no fever and are therefore no longer infectious, usually 3 to 5 days after the onset of clinical manifestations of disease.

### Replication of dengue virus

Mosquito-borne DENV is a single-stranded positive RNA virus which belongs to *Flavivirus* genus from *Flaviviridae* family (reviewed in Screaton et al., 2015 [6]). There are four antigenically different serotypes DENV sharing 60–80% homology: DENV-1, DENV-2, DEN-3 and DENV-4. DENV is a 50-nm virus particle surrounded with a lipid membrane. The genomic RNA of about 11.000 nucleotides of length contains a single open reading frame which encodes a large polyprotein that is co- and post-translationally processed into three structural proteins, capsid (C), precursor membrane (prM) and envelope (E) and seven nonstructural proteins, NS1, NS2a, NS2b, NS3, NS4a, NS4b, and NS5. The E glycoprotein is the major component of the virion surface and the main target of DENV neutralizing antibodies. The ectodomain of E comprises a centrally located domain I (EDI), a domain II (EDII) containing the fusion loop and a domain III (EDIII) which brings most of the serotype-specific antibody epitopes.

Once the genomic RNA is released into the cytoplasm upon the fusion of viral membrane with endosomal membranes, viral RNA translation can start at the close vicinity of the endoplasmic reticulum (ER) membranes. After the viral replication complex is synthesized, translation switches off, and viral RNA synthesis begins. During DENV replication, the viral genome is degraded by exonuclease XRN1 to generate small species of subgenomic flavivirus RNA (sfRNAs) which correspond to a large part of the non-translated genomic (NTR) region at the 3ʹ end of genomic RNA [7]. Accumulation of sfRNAs contributes to viral replication but also alters the host-cell antiviral immune responses to the benefit of virus replication [8]. *De novo* synthesized viral RNA molecules are packaged into nucleocapsids or engaged in massive production of viral proteins. Virus assembly occurs at the ER surface, resulting in non-infectious, immature viral particles. Immature viral particles are transported through the *Golgi* complex and then prM is processed to M by furin-like proteases until the fully mature infectious virus particle is released into the extracellular environment.

### Symptomatic features of dengue disease

The four serotypes of mosquito-transmitted DENV can cause a wide spectrum of clinical manifestations which range from asymptomatic or paucisymptomatic to severe infections. After an incubation period of 3 to 7 days, dengue disease is characterized by three phases (reviewed in details in Simmons et al 2012 [9]):

- Febrile: typically characterized by high temperature (> 38,5°c) accompanied by headache, vomiting, myalgia, and joint pain, sometimes with a transient macular rash;
- Critical: In a small proportion of patients, typically in children and young adults, a systemic vascular leak syndrome becomes apparent around the time of defervescence;
- Recovery: reverting spontaneously to a normal level. A second rash may appear during the recovery phase. Adults may have profound fatigue for several weeks after recovery.

It is now well recognized that the majority of people infected with dengue have no obvious clinical signs (asymptomatic) or are paucisymptomatic. However, some individuals can experience clinical symptoms of disease. Patients with dengue fever who improve after defervescence are diagnosed with a non-severe form of dengue, while others are considered to have severe symptoms. Around the time of defervescence, when the temperature drops to 37.5 to 38°C or less and remains below this level, usually on days 3–7 of illness, an increase in capillary permeability may occur. This phenomenon marks the beginning of the critical phase. The period of clinically significant plasma leakage usually lasts 24–48 hours and the degree of plasma leakage varies among patients. When a critical volume of plasma is lost through leakage, acute peripheral circulatory failure occurs leading to insufficient inflow of oxygen-rich blood to body cells. But this condition of low blood perfusion to tissues resulting in cellular injury and inadequate tissue function called ‘shock’ is often preceded by warning signs. For example, changes in the full blood count should be used to guide the onset of the critical phase and plasma leakage, and this way prevents shock. With prolonged shock, the consequent organ hypoperfusion results in progressive organ impairment, metabolic acidosis and disseminated intravascular coagulation. This in turn leads to severe hemorrhage causing the hematocrit to decrease in severe shock which considerably impairs patient vital prognosis if not cared. As a matter of fact, the case fatality rate in untreated dengue patients was reported to reach up to 20% and reduced to less than 1% under expert clinical management.

Today, no specific antiviral therapies are available for dengue virus infection and clinical management of the more severe forms of disease is mainly based on supportive therapy and intravascular volume replacement [10,11].

### WHO classification guidelines of dengue disease

#### Classification history

For greater clarity on distinctions between classic dengue fever and dengue hemorrhagic fever or severe dengue, a World Health Organization (WHO) committee developed case classification guidelines in 1974 (WHO 1975), based on studies of disease patterns in children in Thailand in the 1960s, which were subsequently modified and published a number of times. The 1997 guidelines (2^nd^ edition) classified dengue into Dengue Fever (DF), Dengue Hemorrhagic Fever (DHF Grades 1 and 2) and Dengue Severe Syndrome (DHF Grades 3 and 4) (WHO 1997). The case diagnosis for DF emphasized the need for laboratory confirmation. The experience of this classification system has highlighted a number of limitations. This classification is based in particular on clinical data collected from Thai children, which may not be universally representative of dengue fever after its expansion into other tropical regions and older age groups. A range of clinical trials requiring repetition is also needed, which may be difficult for countries with limited resources to implement. The tourniquet test, a measure of capillary fragility and thrombocytopenia for the diagnosis of DHF, is an integral part of the 1997 case definitions. However, the test does not have sufficient sensitivity and specificity to effectively differentiate cases of DF and DHF, and dengue fever from other febrile diseases. For all these reasons, a new classification was established in 2009 by WHO.

#### Current classification

The 2009 classification into severity levels is considered to be more sensitive in capturing severe disease than the 1997 guidelines (WHO 2009). The 2009 WHO criteria classify dengue according to levels of severity: dengue without warning signs; dengue with warning signs (abdominal pain, persistent vomiting, fluid accumulation, mucosal bleeding, lethargy, liver enlargement, increasing hematocrit with decreasing platelets); and severe dengue (dengue with severe plasma leakage, severe bleeding, or organ failure). Patients who recover from defervescence are considered to have mild dengue fever, but those whose condition does not ameliorate after this period tend to manifest warning signs. These people are likely to recover with intravenous rehydration. However, further deterioration is classified as severe dengue, though recovery is possible if appropriate and timely treatment is given.

## Human susceptibility to dengue virus infection

### Asymptomatic and symptomatic cases of dengue

It is now well admitted that a majority of dengue infected people are clinically asymptomatic [12]. Unexpectedly, it has been observed that these inapparent infections are potentially capable of infecting *Aedes aegypt*i [13] . Recently, ten Bosch et al. argued that the rapid spread of DENV in endemic areas, as well as enhanced transmission of disease by the invertebrate vectors, could be largely attributable to asymptomatic or paucisymptomatic cases of dengue [14]. Given that the number of dengue cases is probably underestimated, it is a high priority to survey the actual level of dengue endemicity in the regions affected by the disease. Such information would be essential for the introduction of a dengue vaccine as WHO has recommended with the recently commercialized Dengvaxia® vaccine by Sanofi-Pasteur.

People with clinically apparent DENV infection may develop severe dengue requiring hospitalization. The exact causes of severe dengue still remain elusive. The etiology of severe dengue is undoubtedly multifactorial with host factors and immune status having important roles [15–17]. The question of the existence of biological markers of severe dengue as prognostic signatures of the risk of evolution of disease was addressed [18,19]. Nhi et al. identified plasma proteins exhibiting different relative concentrations in patients diagnosed for a severe dengue [20]. Nikolayeva et al. worked on a small number of gene expression markers to detect severe cases of dengue fever from blood samples from young patients with secondary infection [21]. Once the biological prognostic markers for severe dengue fever are identified, their validation will require large and independent cohorts of dengue patients who differ in ages and immune status. Regarding the pathogenic properties of DENV, it has been widely reported that DENV-2 infection causes a greater number of severe cases than other serotypes [22].

### Antibody response to dengue virus infection

We now know that humoral immunity plays an important role in eliminating DENV infection and protecting against disease. The effective response of anti-dengue antibodies, stimulated in response to primary DENV infection, lasts about 2 years, after which the individual is susceptible to re-infection with another serotype.

Neutralization of virus infectivity by antibodies is assumed to be the key mechanism by which protection against DENV is achieved [18,23]. The major target of DENV neutralizing antibody is the E protein, even though anti-M and anti-NS1 antibodies have also been shown to be protective [24,25]. The free viral particles are bound by a diverse set of neutralizing and non-neutralizing antibodies directed against multiple different antigenic sites, and to the epitopes of these sites, which act together to effect neutralization. Infection can be prevented by antibodies through various mechanisms including: blocking of binding, inhibition of fusion of the viral membrane with the endocytic vacuole membrane which inhibits the release of viral RNA into the cytoplasm, and lysis of the antibody-coated virus by the complement.

However, specific antibodies against DENV could be associated with the development of severe dengue. Indeed, it has been proposed that infected people with a low neutralizing antibody titer are at a higher risk of severe dengue compared to those with titers above 100 [12]. Severe dengue is associated with secondary infections, suggesting that pre-existing immunity might play a key role in dengue pathogenesis [26]. A hallmark of secondary dengue fever is a more rapid and higher antibody response than the primary response. This is caused by the stimulation of memory B-cells from the primary infection. In fact, in this case, the antibodies produced as a result of the secondary infection remain more fitted to neutralize the serotype responsible for the primary infection than the current infecting virus. This phenomenon has been called ‘Antibody-Dependent Enhancement’ (A.D.E) or ‘Original Antigenic Sin’ as a dominance of B-cell response to primary DENV infection [27]. As such, A.D.E. could enhanced the pathogenicity of DENV infection or additional inflammatory responses, and could also significantly alter vaccine efficacy (to be discussed later) [28]. In secondary infection, the neutralizing antibody response extends over time. A key feature of secondary dengue fever is a durable response that neutralizes or sub-neutralizes multiple serotypes, including those that have not yet infected the individual. Cases of tertiary or quaternary DENV infections have rarely been documented, which supports the notion that secondary infections may stimulate a long-term cross-neutralizing antibody response that may be effective.

## Dengue vaccine development

### The current challenges for dengue vaccine development

The global spread of dengue disease is mainly linked to urban population growth in endemic areas, climate change, globalization of economic exchanges and intensification of intercontinental travels [11]. Such factors largely facilitated the hyperendemicity of dengue in Latin America and South-East Asia, and contributed to intensive circulation of different dengue serotypes through the tropical regions too [29]. The majority of dengue infections are clinically asymptomatic and inapparent infections may significantly contribute to the burden of disease in endemic areas [14].

An efficient dengue vaccine is expected to afford a rapid and high protection level against all circulating DENV serotypes regardless of individual immune status and age of vaccination. Furthermore, such a vaccine should be efficient in producing long-term, type- or cross-specific neutralizing antibodies against each of the four DENV serotypes. Lastly, neutralization titers needed for protection should be higher than those usually accepted for other flavivirus vaccines such as yellow fever and Japanese encephalitis. Serotype-specific and cross-reactive CD8 T cells could contribute to the long-term immune protection against DENV infection [30]. Although induction of neutralizing antibodies may play a pivotal role in dengue vaccination, recent data argue in favor of the development of dengue vaccine candidates that also induce protective CD8^+^ T cell response [31–33].

Initially, the development of dengue vaccines was hampered by a series of major obstacles. First, the evolution of virus strains within the four dengue serotypes and their genotypes is often rapid and unpredictable, intensifying the inherent difficulties associated with developing a dengue vaccine candidate. Secondly, the development of effective vaccines against dengue fever remains hindered in the absence of an accurate animal model mimicking the pathogenesis of dengue virus infection in humans, combined with a clear lack of knowledge concerning the major risk factors of severe outcome [11,34,35]. Advances have been recently made on development of appropriate animal models – immunocompromised mice deficient in type-I and -II interferon receptors and the non-human primates (NHP) – suitable for assessing the protective activity of dengue vaccines [36–40]. However, the use of animals and, in first instances, NHPs for biomedical research represents a serious ethical dilemma especially in the European Union. Their progressive substitution by *in vitro* biological tests is a topical issue.

Considering the important genetic variations that exist between the different serotypes, as well as viral strain diversity within each serotype, a dengue vaccine candidate is expected to induce a balanced and efficient immune response against the different genotypes from the four serotypes [10,34,41]. Such a challenge highlights a first major constraint in the formulation of dengue vaccine. A second important issue relates to the adverse effects associated to A.D.E. phenomenon occurring on secondary infection [10,41–44]. Briefly, the enhanced severity of dengue disease in individuals experiencing a heterologous secondary infection could be due to A.D.E caused by non-protective heterotypic antibodies arising from the primary infection [10,11,34,41–44]. Although *in vivo* evidence of the phenomenon is still lacking, one of the chief concerns is that a dengue vaccine candidate must be able to stimulate simultaneously a sterilizing immunity against all serotypes. A possible A.D.E between members of flavivirus genus such as Zika virus (ZIKV) and DENV has been suggested due to the antigenic cross-reactivity of dengue antibodies with contemporary epidemic strains of ZIKV [45]. Whether dengue vaccination can have beneficial or detrimental impacts on ZIKV infection due to strong antigenic cross-reactivity between ZIKV and DENV is an important issue to be taken into account in the development of dengue vaccines [42,46,47].

Which remarkable advances have been recently made toward the development of effective dengue vaccines? Several dengue vaccine candidates are in the pipeline. There are live-attenuated and inactivated viruses, DNA constructs, vector-based expression, virus-like particles and recombinant subunit antigens [48–50]. In 2016, the first commercial dengue vaccine called Dengvaxia® has been registered in several endemic dengue countries including Mexico, Brazil, and Philippines [44,51]. Dengvaxia®, as well as the most advanced candidate vaccines are discussed below.

### Current status of dengue vaccine development

The development of effective tetravalent dengue vaccines is a high priority especially in regions infested by the mosquito vectors of the disease [10,29,52,53]. The first tetravalent dengue vaccine CYD-TDV (Dengvaxia® developed by the manufacturer Sanofi-Pasteur) has been recently licensed in Asia and Latin America. The efficacy of CYD-TDV was evaluated on the basis of large phase 3 clinical trials where young children had been included. Two additional live-attenuated dengue vaccines are in phase 3 development: the TDV developed by Takeda and the *LAV Delta 30* developed by NIH/Butantan Institute. Today, the two live-attenuated vaccines (LAV) for dengue, TDV and *LAV Delta 30*, are the two most advanced candidates in clinical studies.

### Dengue vaccine Dengvaxia®

**Figure 2.**
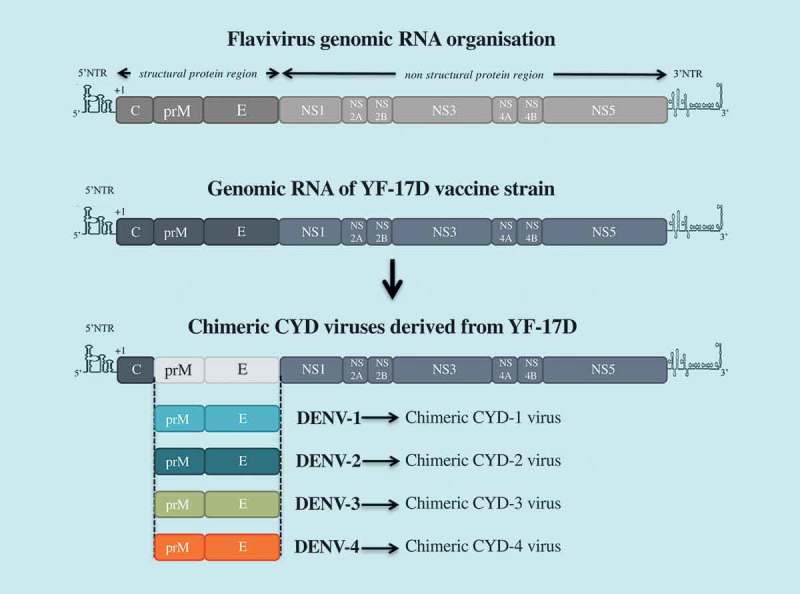
CYD-TDV (Dengvaxia®) vaccine. The tetravalent dengue vaccine CYD-TDV has been constructed by substituting prM and E genes from DENV-1 to DENV-4 into the backbone of the molecular clone of yellow fever vaccine YF-17D. there are four chimeric CYD-1 to CYD-4 which compose the CYD-TDV.

It is widely accepted that dengue vaccination should induce a balanced protective immunity against all four serotypes [34,41]. Based on this premise, the chimeric tetravalent dengue vaccine CYD-TDV has been generated using a flavivirus vaccine – the live-attenuated 17D strain of yellow fever virus (YF-17D) – as a backbone (Figure 2). CYD-TDV contains four chimeric YF-17D viruses which express the prM and E proteins from each of the four dengue serotypes [43,44]. Started in 2011, the vaccine candidate combined multi-center pivotal Phase 3 clinical trials with tens of thousands of individuals in different regions of Asia and Latin America following the same protocol [51,54]. Neutralization antibody titers higher than 1:10 have been accepted as a correlate of protection against dengue disease. In 2018, the manufacturer Sanofi-Pasteur has already licensed CYD-TDV vaccine (Dengvaxia®) for dengue prevention in twenty endemic countries worldwide. Three doses of the vaccine are administered to recipients at 0, 6 and 12-month intervals.

Clinical studies showed that Dengvaxia® efficacy is less than 70% among recipients. The vaccine has been shown to be poorly effective against DENV-2, which is however the main serotype in terms of medical problems. Moreover, the efficacy of Dengvaxia® significantly varied according to the age and immune status of an individual [55,56]. During the four years of the phase 3 clinical trial, an unexpected number of hospitalizations for clinical dengue infection was significantly observed among vaccinated participants not older than 9-years-old [51]. The risk of hospitalization for severe dengue was mostly observed after inoculation of the first dose of Dengvaxia® [56]. The mechanism explaining this increased risk of dengue disease in the younger recipients of Dengvaxia® is not yet understood. Clinical studies have shown that higher vaccine efficacy has been reported among individuals with pre-existing immunity to flaviviruses, suggesting that natural infection may contribute to establishing long-term adaptive immunity against dengue fever [54]. *A contrario*, vaccine recipients who had not been previously exposed to dengue or having a limited anti-dengue immunity manifested a higher risk of severe dengue upon vaccination with Dengvaxia® [10,57]. The manifestation of A.D.E. due to a too low antibody titer and/or differences in vaccine efficiency according to the four infecting serotypes could explain the increased risk of severe dengue fever in vaccinated subjects without prior experience of DENV infection [57]. Despite all these restrictions, Sanofi-Pasteur considers Dengvaxia® to be a beneficial vaccine given the lower incidence of severe dengue and the number of people hospitalized in the various endemic countries where it has been introduced [57–60].

In April 2018, WHO published the latest recommendations of the Strategic Advisory Group of Experts on Immunization (SAGE) on the use of Dengvaxia® vaccine in dengue endemic areas (WHO Weekly Epidemiological Record, Friday 8 June 2018). To date, SAGE recommends the deployment of Dengvaxia® vaccine commercialized by Sanofi-Pasteur only in endemic regions where dengue seroprevalence is higher than 70% among the target population, excluding individuals under 9 years of age. The age limit for vaccination would depend on the level of dengue transmission in the area concerned, but is generally 45 years. To prevent adverse events related to the immunological status of vaccinated individuals, strong recommendations have been made to implement a ‘pre-vaccination screening’ strategy in countries where it is necessary to assess population exposure to dengue fever or where the expected transmission rate is low. To this purpose, Sridhar et al. recently developed a new diagnostic test based on DENV NS1 that was performed on blood samples taken from individuals one year after receiving a single dose of Dengvaxia®[57].

## Dengue vaccine candidate TDV

**Figure 3.**
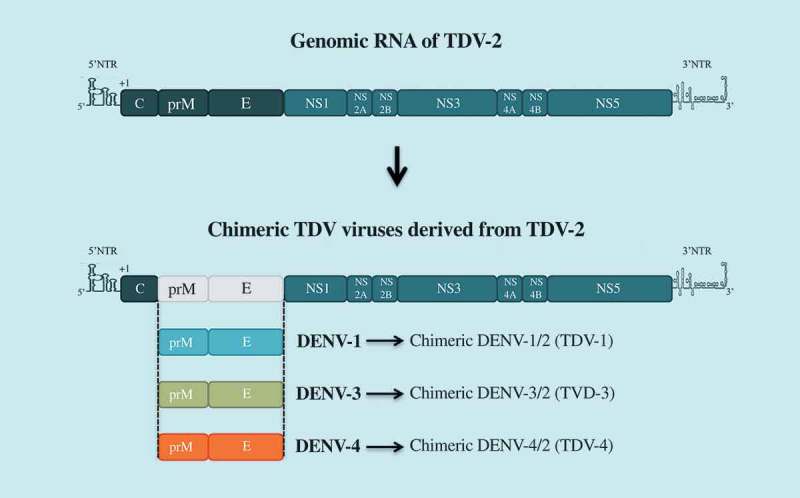
TDV vaccine candidate. The Takeda tetravalent vaccine TDV has been generated by substituting prM and E genes from DENV-1, DEN-3, and DENV-4 into TDV-2 backbone. TDV2 is an over-attenuated mutant of DENV-2 vaccine candidate DENV-2-PDK53.

Takeda’s tetravalent dengue vaccine (TDV) candidate is based on mutant TDV-2 derived from the original vaccine strain DENV-2-PDK-53, over-attenuated by the introduction of an additional mutation in NS3 [61–63],(Figure 3). Three chimeric viruses TDV-1, TDV-3, and TDV-4 have been constructed by substituting prM and E gene from DENV-1, DENV-3 or DENV-4 into the TDV-2 backbone [64,65]. In clinical studies conducted during phases 1 and 2, TDV was reported as a safe and well-tolerated vaccine candidate that induces neutralizing antibody and CD8^+^ T cell responses against all four serotypes [66–68]. Another phase 1b study was performed to investigate the immunogenicity of early low-doses of TDV (LD-TDV) following intradermal administration in healthy dengue-naive adults [69]. The administration of two doses of DL-TDV, 3 months apart, induces immune responses against the four dengue serotypes, the highest being DENV-2 and the lowest DENV-4. TDV vaccine recipients had neutralizing antibodies against DENV directed to the EDIII antigenic domain of DENV-2 or EDI of DENV-1 [70]. A two-dose regimen, administered 3 months apart, was selected for the ongoing phase 3 efficacy trial, enrolling about 20,000 individuals aged 4 to 16 years in different countries in Latin America and Asia. Phase 3 is expected to be completed by the end of 2021.

### Dengue vaccine candidate LAV Delta 30

**Figure 4.**
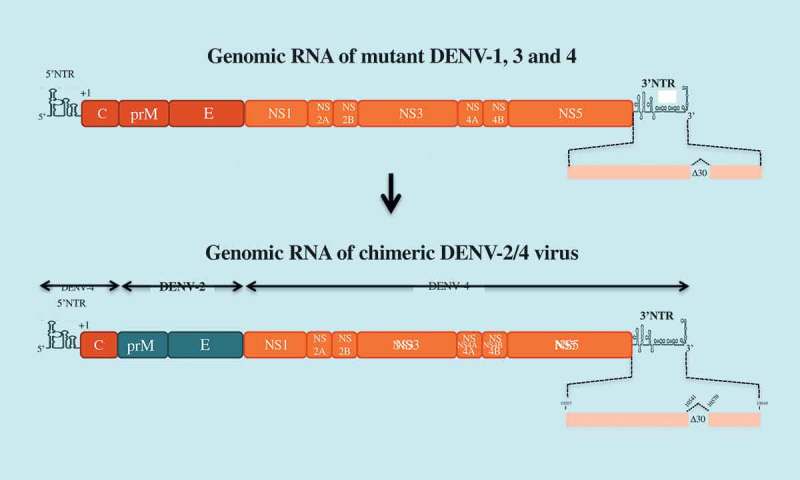
*LAV Delta 30* vaccine candidate. The tetravalent dengue vaccine *LAV Delta 30* (or TV003) is composed of four mutants DENV-1 to DEN-4 which contain a deletion of 30 nucleotides (∆30) in the domain II of the 3ʹNTR of their genomic RNA. mutant DENV-4 virus ∆30 was used as a backbone to generate a chimeric DENV-2 virus ∆30 by substitution of the prM and E genes.

National Institute of Health (NIH) has developed a tetravalent dengue candidate vaccine, *LAV Delta 30*, which contains a deletion of 30 contiguous nucleotides (∆30) into the domain II from the 3ʹ end NTR of the genomic RNA. The ∆30 deletion has been used to attenuate DENV-1, −3, and −4 viruses (Figure 4)[71]. A chimeric DENV-2 virus containing the prM and E of DENV-2 in the context of mutant DENV-4 virus ∆30 was also generated. The marked susceptibility of *LAV Delta 30* to the antiviral action of type-I interferon may explain the attenuated properties of the LAV due to reduced accumulation of sfRNAs in infected cells [72]. *LAV Delta 30* was licensed to Butantan Institute to produce a tetravalent vaccine known as ‘TV003’. This vaccine shares an equal dose of each recombinant DENV of the four serotypes. Studies conducted in phase 1 and 2 demonstrated the safety and immunogenicity of TV003, with seroconversion rates ranging from 50% (DENV-2) to 100% (DENV-1, 3, and −4) after a single dose. However, viremia was still detectable in individuals vaccinated with TV003 [73]. Up to 90% of vaccine receivers developed neutralizing antibodies after a single dose. TV003 vaccination provided protection against a challenge with DENV-2 one year later [74]. A boost of vaccine recipients with a second dose of TV003 significantly increased the seroconversion rate against DENV-2 [74]. Vaccine candidate TV003 is currently in Phase 3 clinical trial which would be completed in 2022.

### Dengue vaccine involving DNA technology

Another variety of current dengue vaccine candidates involves recombinant DNA technology. There are recombinant subunit vaccines requiring expression systems such as plasmid DNA, heterologous vectors, virus-like particles based on the co-expression of the prM and E proteins or only displaying EDIII of the four serotypes, and purified recombinant viral antigens [34,43,47–50,75–77]. Most dengue vaccine candidates are in preclinical studies or still in Phase I clinical trials.

## Discussion

Vaccination on the human population is one of the key strategies to prevent the risk of dengue virus transmission from a human host to a mosquito vector [52,53]. The increasing incidence of co-infection with all four serotypes underlines the urgent need for a tetravalent dengue vaccine. This vaccine must be capable of inducing long-term protective immunity against all four dengue serotypes, regardless of the age of the vaccine recipient or their dengue serostatus at the time of vaccination. Dengvaxia® is the first tetravalent vaccine licensed against dengue that has been approved in about 20 countries in Asia and Latin America. WHO recently imposed severe restrictions on the use of Dengvaxia®, seriously calling into question the continuation of mass dengue vaccination programs in dengue-endemic countries around the world. At the end of 2017, vaccination with Dengvaxia® was suspended in the Philippines due to adverse events in the vaccinated population. Validation of other dengue vaccination strategies is therefore a high priority. The new vaccines must ensure indisputable efficacy in protecting against all dengue serotypes and circulating viral strains regardless of the age and immunological status of those at risk of infection. Currently, TDV and *LAV Delta 30* are the two most advanced alternatives to CYD-TDV. Nevertheless, the final verdict remains to be pronounced as the evaluation of the protective efficacy linked to each strategy is still in progress. As an alternative to the *LAV* strategy, the Walter Reed Army Research Institute (WRAIR), FIOCRUZ and GlaxoSmithKine (GSK) recently joined forces in the development of a dengue purified tetravalent inactivated vaccine (DPIV) that has been administered in two separate doses to a group of adults [78]. Adjuvant formulations were supplied by the manufacturer, GSK. Results from the Phase 1 clinical study showed that adjuvanted DPIV is well tolerated and immunogenic, justifying the initiation of a Phase 2 trial in the near future.

Intriguing changes in human susceptibility to dengue disease have been recently documented in different endemic regions. So far, the classic pattern of dengue fever endemicity is that the pediatric population was most likely to experience dengue infection due to the absence of protective immunity. However, recent epidemiological data would tend to clearly nuance this assertion [79]. A set of systematic reviews based on outbreaks from 2000 pointed out a gradual shift in the burden of dengue from the children to the adult population among five of the seven hyperendemic regions [58,80–85]. A large systematic review and meta-analysis including 96 studies reporting outbreaks from 1999 to 2015 in all seven WHO regions was published [2]. In this work, Guo *et al*. confirmed this trend and reported that it became more pronounced in the last 5 years. Indeed, they described that the mean age of infected people for this period was around 30 years, and increased to 34 years in outbreaks occurring later than 2010. Recently, cases of serious dengue fever have been reported in Puerto Rico, Singapore and Bangladesh highlighting that the adage of the adult population being at low risk of severe dengue outcomes may no longer be true [86–88]. In the current epidemic of DENV-2 in La Reunion island (Indian ocean) that started in early 2018 [89], most hospitalized cases of dengue disease are people aged 15 to 65, while only a small number of pediatric hospitalizations have been reported to date. Furthermore, studies of dengue disease in the elderly have already warned about the increasing burden of disease in this population. Indeed, it has been shown that the elderly present atypical clinical features with fewer symptoms that could lead to misdiagnosed dengue cases [90,91]. Combined with a higher risk of co-morbidities and an increased case-fatality rate, there is a need to refine diagnostic criteria for reliable classification and assessment of the risk of progression of a severe disease [92]. Such changes in epidemiological trends challenge the current management strategies and raise the need to adjust and/or develop new vaccination strategies more appropriate to such a target population.
